# Towards population-independent, multi-disease detection in fundus photographs

**DOI:** 10.1038/s41598-023-38610-y

**Published:** 2023-07-17

**Authors:** Sarah Matta, Mathieu Lamard, Pierre-Henri Conze, Alexandre Le Guilcher, Clément Lecat, Romuald Carette, Fabien Basset, Pascale Massin, Jean-Bernard Rottier, Béatrice Cochener, Gwenolé Quellec

**Affiliations:** 1 Université de Bretagne Occidentale, Brest, Bretagne, France; 2 INSERM, UMR 1101, Brest, F-29 200 France; 3 IMT Atlantique, Brest, F-29200 France; 4 Evolucare Technologies, Villers-Bretonneux, F-80800 France; 5 Service d’Ophtalmologie, Hôpital Lariboisière, APHP, Paris, F-75475 France; 6 Bâtiment de consultation porte 14 Pôle Santé Sud CMCM, 28 Rue de Guetteloup, Le Mans, F-72100 France; 7 Service d’Ophtalmologie, CHRU Brest, Brest, F-29200 France

**Keywords:** Diseases, Health care, Medical research

## Abstract

Independent validation studies of automatic diabetic retinopathy screening systems have recently shown a drop of screening performance on external data. Beyond diabetic retinopathy, this study investigates the generalizability of deep learning (DL) algorithms for screening various ocular anomalies in fundus photographs, across heterogeneous populations and imaging protocols**.** The following datasets are considered: OPHDIAT (France, diabetic population), OphtaMaine (France, general population), RIADD (India, general population) and ODIR (China, general population). Two multi-disease DL algorithms were developed: a Single-Dataset (SD) network, trained on the largest dataset (OPHDIAT), and a Multiple-Dataset (MD) network, trained on multiple datasets simultaneously. To assess their generalizability, both algorithms were evaluated whenever training and test data originate from overlapping datasets or from disjoint datasets. The SD network achieved a mean per-disease area under the receiver operating characteristic curve (mAUC) of 0.9571 on OPHDIAT. However, it generalized poorly to the other three datasets (mAUC < 0.9). When all four datasets were involved in training, the MD network significantly outperformed the SD network (*p* = 0.0058), indicating improved generality. However, in leave-one-dataset-out experiments, performance of the MD network was significantly lower on populations unseen during training than on populations involved in training (*p* < 0.0001), indicating imperfect generalizability.

## Introduction

With growing and aging populations, automatic screening of ocular anomalies in fundus photographs is a promising solution to scale-up screening and face the shortage of trained experts (ophthalmologists, retina specialists). It has shown significant progress in recent years, especially with the breakthrough of deep learning (DL). The first automated algorithms targeted screening of a vision threatening pathology, diabetic retinopathy (DR)^1,2^. This is mainly thanks to the large amount of annotated data which have been collected and labeled with clinical diagnosis and severity by experts in teleretinal screening programs^3,4^. Currently, many automated DR screening algorithms have shown performances comparable to, or even better than, human experts^5,6^. In addition, many algorithms have been implemented in practice as clinical devices for screening DR: RetinaLyze (RetinaLyze System A/S, Copenhagen, Denmark), IDxDR (Digital Diagnostics, Coralville, IA, USA), RetmarkerDR or Retmarker Screening (RETMARKER S.A., Coimbra, Portugal), EyeArt (Eyenuk, Woodland Hills, CA, USA), and OphtAI (OphtAI, Paris, Île-de-France, France). Among these cited devices, IDx-DR and EyeArt have U.S. Food and Drug Administration (FDA) approval for detecting DR levels more severe than mild and without the requirement for further interpretation by a clinician^7,8^. These tools were generally developed using a large dataset coming from a diabetic population. They have demonstrated to be highly accurate using different retinal camera models, imaging protocols, and across multiple ethnicities^5,6,9,10^. Nevertheless, the lack of external validation in real world settings where accuracy is likely to be reduced due to changes in disease frequency, image quality and patient characteristics has been considered a prominent issue. To address this issue, prospective evaluation studies^2,11,12^ have been proposed to validate an artificial intelligence-enabled DR screening algorithm on real data. In addition, a more recent study compared the performances of seven automated AI-based DR screening algorithms against human graders on real data^13^.

However, one limitation of the aforementioned studies is that DL systems have only been validated for classification of a single eye disease. Since then, DL algorithms have also been developed to screen for other specific pathologies such as glaucoma^14–18^, age-related macular degeneration (AMD)^19–22^, cataract^23^ and degenerative myopia^24^. Even more, researches have progressed to include the detection of multiple ocular diseases^25–27^. For instance, multiple challenges were organized for multi-disease automatic detection^28,29^. However, algorithms competing in these challenges were developed and tested on a specific dataset coming from a particular population. On the other hand, Son et al.^25^ assessed the generalization of their automated multi-disease algorithm on different datasets (the Indian Diabetic Retinopathy image Database (IDRiD) and e-ophtha). However, the comparison was done on diabetic datasets and on limited number of abnormalities: 3 abnormalities for the IDRiD dataset and 2 abnormalities for the e-ophtha dataset.

Despite these advancements, no study has yet validated an automated multi-disease screening algorithm in a scenario where the test data is very different from the training data. This is the purpose of this study: we evaluate state-of-the-art DL algorithms in scenarios where training and test data come from different populations, were acquired with different cameras and were annotated following different protocols. The following datasets are considered: OPHDIAT (France, diabetic population)^3^, OphtaMaine (France, general population)^30,31^, RIADD (India, general population)^32^ and ODIR (China, general population)^33^. The following diseases are targeted: diabetes (D), glaucoma (G), cataract (C), AMD (A), hypertension (H), myopia (M) and other diseases/abnormalities (O). Note that each dataset was initially labeled for a different set of ocular anomalies, with its own taxonomy: for the purpose of this study, ground-truth annotations have been unified retrospectively according to the ODIR annotation class system^33^.

**Figure 1:**
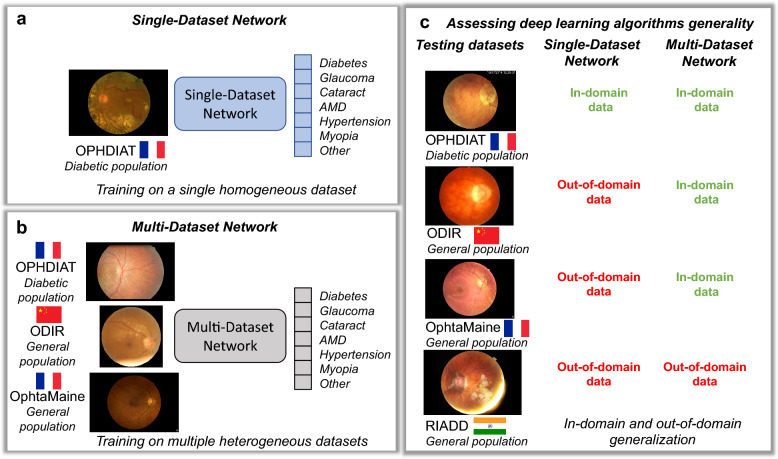
An overview of our proposed study. (**a**) A single-dataset network trained on a single homogeneous dataset. (**b**) A multi-dataset network trained on multiple heterogeneous datasets. (**c**) Assessing deep learning algorithms generality for data coming from an in-domain or out-of-domain distribution.

We hypothesize that training a DL algorithm jointly on multiple datasets, from distinct populations, will result in improved generality compared to a DL algorithm trained on a single dataset. To challenge this hypothesis, two scenarios are considered in this study. In a first scenario, inspired by the commercially available DL solutions, the DL algorithm is trained on a large dataset collected from a diabetic population screened for DR, namely OPHDIAT^3^. In a second scenario, the DL algorithm is trained on multiple datasets simultaneously. The resulting DL networks are referred to as Single-Dataset (SD) network and Multiple-Dataset (MD) network, respectively. In both scenarios, we investigate classification performance whenever training and test data originate from overlapping populations (although different patients) or from disjoint populations. Our proposed pipeline is presented in Fig. 1.

## Results

A total of 77,827 images from OPHDIAT, 17,120 images from OphtaMaine, 3,200 images from RIADD and 10,000 images from ODIR were included in this study. Each of these datasets was divided into a training, a validation and a test subset: the characteristics of these subsets are detailed in Table 1.

**Table 1:** Frequency of each ODIR category in the four considered datasets: ODIR $(I$), OPHDIAT ($P$), OphtaMaine $(A$) and RIADD ($R$).

	N	D	G	C	A	H	M	O
ODIR								
$I$	3248 (1624)	3240 (1620)	610 (305)	616 (308)	476 (238)	298 (149)	486 (243)	2786 (1393)
${I}_{Train}$	2276 (1138)	2260 (1130)	430 (215)	424 (212)	328 (164)	206 (103)	348 (174)	1964 (982)
${I}_{Validation}$	324 (162)	326 (163)	64 (32)	62 (31)	50 (25)	32 (16)	46 (23)	272 (136)
${I}_{Test}$	648 (324)	654 (327)	116 (58)	130 (65)	98 (49)	60 (30)	92 (46)	550 (275)
OPHDIAT								
$P$	16,955	30,065	10,624	3541	3173	3018	1209	13,380
${P}_{Train}$	13,708	24,321	8684	2834	2579	2437	964	10,905
${P}_{Validation}$	1557	2714	891	363	287	274	106	1200
${P}_{Test}$	1690	3030	1049	343	307	307	139	1275
OphtaMaine								
$A$	14,785 (7104)	86 (33)	825 (393)	4 (2)	50 (28)	0 (0)	38 (19)	1372 (570)
${A}_{Train}$	5935 (2856)	29 (13)	325 (158)	0 (0)	18 (10)	0 (0)	14 (7)	531 (220)
${A}_{Validation}$	1452 (701)	12 (4)	82 (39)	3 (1)	5 (4)	0 (0)	4 (2)	150 (62)
${A}_{Test}$	7398 (3547)	45 (16)	418 (196)	1 (1)	27 (14)	0 (0)	20 (10)	691 (288)
RIADD								
*R*	669	632	445	523	169	9	167	1591
${R}_{Train}$	401	376	282	317	100	3	101	974
${R}_{Validation}$	134	132	72	102	38	3	34	310
${R}_{Test}$	134	124	91	104	31	3	32	307

For OphtaMaine and ODIR, the frequency of each category is represented as number of images (number of examinations). N: Normal, D: diabetes, G: glaucoma, C: cataract, A: AMD, H: hypertension, M: myopia and O: other diseases/abnormalities.

When reporting the results hereafter, we only consider the disease categories containing at least 10 test images (for OPHDIAT and RIADD) or 10 test examinations (for ODIR and OphtaMaine). Therefore, results for the Hypertensive and Cataract categories, in OphtaMaine, and for the Hypertensive category, in RIADD, were discarded.

For a fair comparison between the SD network and the MD network, the same backbone “tf_efficientnet_b5_ns”^34^ and hyperparameters were used for both networks. These hyperparameters were chosen after a thorough examination as detailed in the Method Section.

### ROC analysis

To assess the generalizability of both networks, out-of-domain testing for multi-disease detection was performed for the SD network and for the MD network. Table 2 reports the mean per-class area under the receiver characteristic curve (mAUC) on the four test subsets, both for the SD network and for the MD network, trained either using $K=3$ training and the corresponding $K=3$ validation (denoted as training/validation) subsets (leave-one-dataset-out) or using all training/validation subsets ($K=4$). The results show that all networks performed well on test subsets coming from one of the populations involved in training. However, their performances were poorer when tested on data coming from a previously unseen population. For instance, on the OPHDIAT test subset:

- the mAUC was 0.9571 for the SD network trained on OPHDIAT,
- the mAUC obtained with $K=3$ training/validation subsets, when leaving OPHDIAT out, was 0.8433,
- the worst mAUC obtained with $K=3$ training/validation subsets, when including OPHDIAT, was 0.9363,
- the mAUC obtained with all $K=4$ training/validation subsets was 0.9409.

**Table 2:** mAUC on the test subset of each dataset for the SD network and the MD network.

	OPHDIAT	OphtaMaine	RIADD	ODIR
SD: OPHDIAT	0.9571	0.8969	0.8744	0.8651
MD ($K=3$): OphtaMaine, ODIR, RIADD	**0.8433**	0.9337	0.9326	0.9055
MD ($K=3$): OPHDIAT, ODIR, RIADD	0.9405	**0.8663**	0.9269	0.9109
MD ($K=3$): OPHDIAT, OphtaMaine, ODIR	0.9363	0.9425	**0.8771**	0.8961
MD ($K=3$): OPHDIAT, OphtaMaine, RIADD	0.9387	0.9369	0.9335	**0.8459**
MD ($K=4$)	0.9409	0.9386	0.9429	0.9012

We indicate in the first column the datasets used for training. On each test subset, bold numbers show the mAUC of the MD network when the associated training/validation subsets are left out and underlined numbers show the mAUC corresponding to the worst mAUC obtained when the associated training/validation subsets are included for training.

Similar observations were found when reporting the results on the OphtaMaine, RIADD and ODIR test subsets.

**Figure 2:**
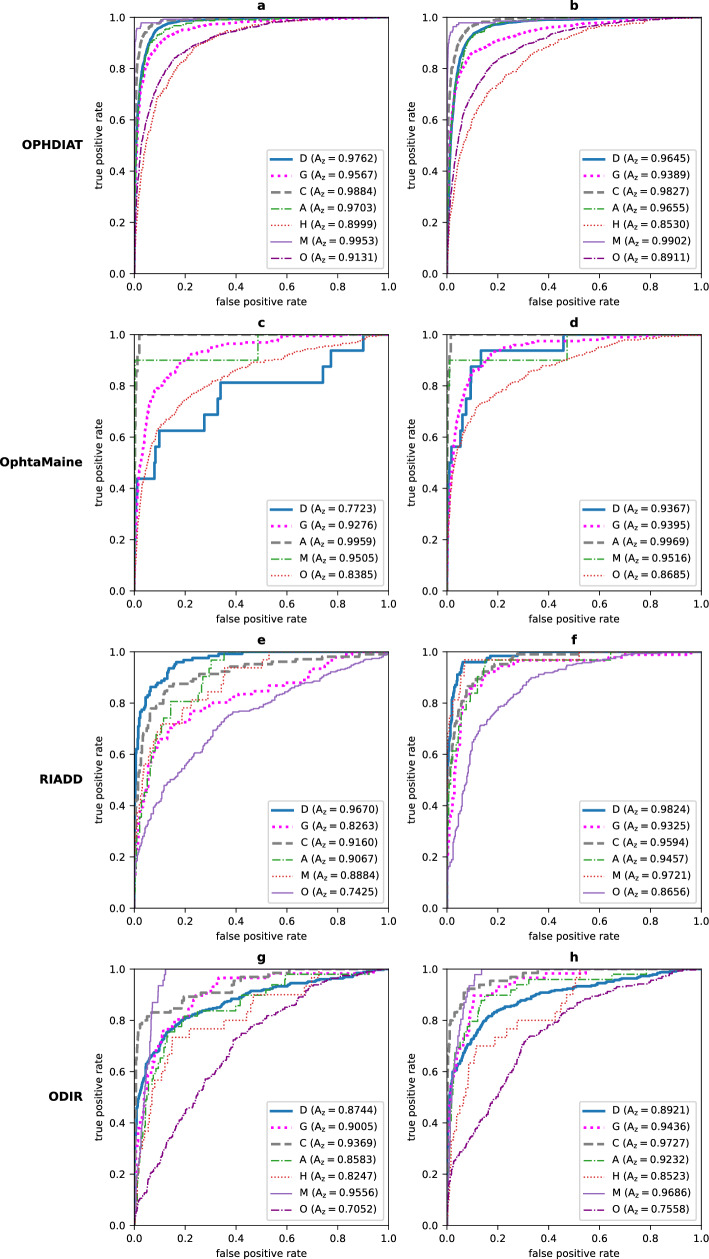
ROC curves for the SD network and the MD network trained on all the datasets ($$K=4).$$ The left column shows the ROC curves for the SD network and the right column shows the ROC curves for the MD network trained on all the datasets ($$K=4)$$ on the OPHDIAT test subset (**a, b**), the OphtaMaine test subset (**c, d**), the RIADD test subset (**e, f**) and the ODIR test subset (**g, h**).

We hypothesized that the MD network, trained using the four datasets, would show superior performance compared to the SD network. To this end, we compared the Receiver Operating Characteristic (ROC) curves of the SD network and of the MD network trained using all training/validation subsets (K = 4) on the four test subsets, in Fig. 2. As shown in Fig. 2, on the OphtaMaine, RIADD and ODIR test subsets, the MD network performed better than the SD network: the AUCs for detecting any category in the ODIR annotations class system were higher for the MD network. However, the performances slightly decreased on the OPHDIAT test subset.

**Figure 3:**
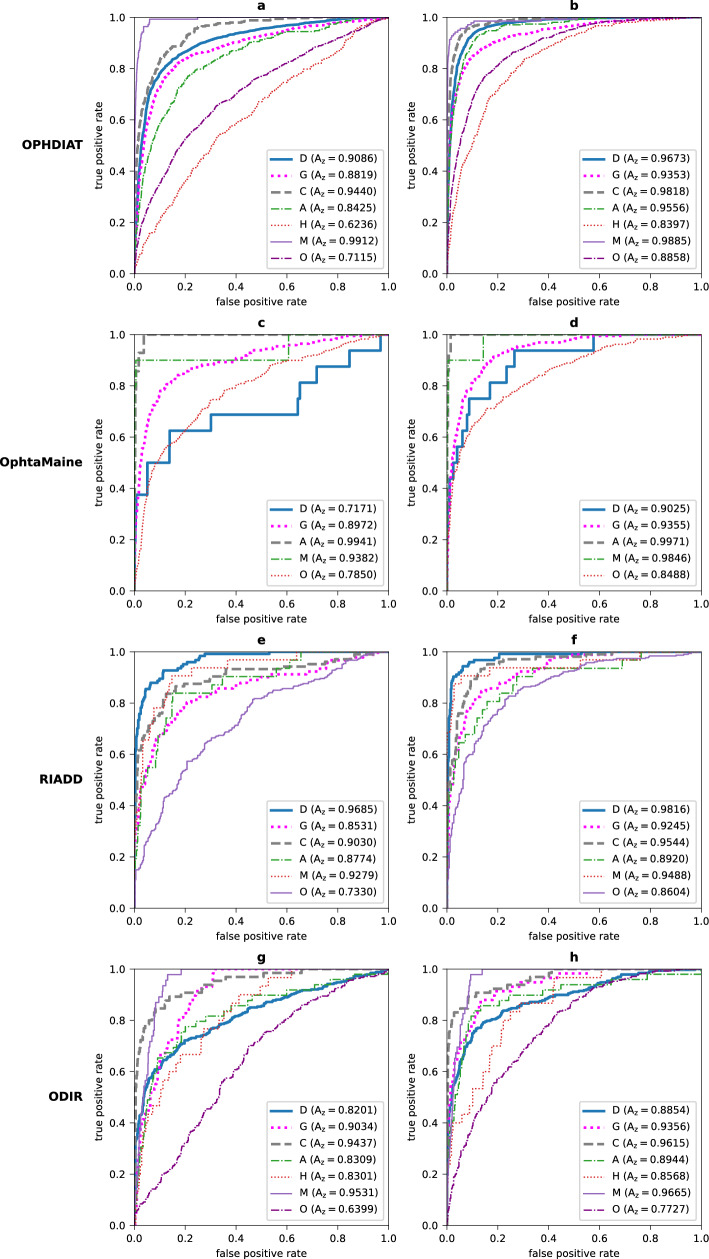
ROC curves for the MD network when a dataset is included for training and when it is left out. On each test subset, the left column shows the ROC curves for the MD network when the associated training/validation subsets are left out and the right column shows the ROC curves when the associated training/validation subsets are included for training the MD network. The ROC curves are shown on the OPHDIAT test subset (**a, b**), the OphtaMaine test subset (**c, d**), the RIADD test subset (**e, f**) and the ODIR test subset (**g, h**).

For assessing the generalizability of the MD network, we compared the performances of the MD network when a dataset is included for training and when it is left out. In Fig. 3, on each test subset, we compare the ROC curves of the MD network when the associated training/validation subsets are left out and of the MD network that corresponds to the worst mAUC obtained when the associated training/validation subsets are included for training. As shown in these figures, the performances of the MD network were better when including the considered dataset in the training subset: the AUCs for detecting pathologies increased notably.

### Statistical test analysis

Table 3 reports a statistical analysis to compare the performances of the SD network and of the MD network. It also compares the performances of the MD network when the training/validation subsets are included for training and when they are left out. The analysis relies on a paired samples Wilcoxon test^35^ to identify whether there is a significant difference between paired samples of AUCs (see Methods for details). This table shows that the differences in AUCs were statistically significant: the null hypothesis was rejected for all data pairs as $p<$ 0.05. Number of positive differences in this table were assigned to data pairs that represent increases in AUCs from sample 1 to sample 2. Number of negative differences were assigned to the opposite case. In both scenarios, it can be concluded that there is a significant decrease in the AUCs when the test data comes from a previously unseen population.

**Table 3:** Summary of Wilcoxon paired test results.

(Sample1)–(Sample2)	Large sample test statistic Z	Two-tailed probability	Number of positive differences	Number of negative differences
SD–MD ($$K=4)$$	− 2.7579	$$p= 0.0058*$$	18	7
SD–MD ($K=3,$ train/validation included)	− 2.300	$p= 0.0214*$	17	8
MD ($K=3,$ train/validation left out)-MD ($K=3,$ train/validation included)	− 4.345	$p<0.0001*$	24	1

*Statistically significant change.

## Discussion

The generalizability of automated, multi-disease screening algorithms is key to deploy them in real-world applications. Most existing deep learning (DL) systems have only been validated for classification of a single eye disease. These algorithms are typically developed using one large dataset, coming from one specific population. In this study, we developed a unified state-of-the-art DL algorithm for automatic detection of multiple anomalies in fundus photographs (the SD network) using data from a single dataset, the OPHDIAT training dataset (France, diabetic population). We assessed the generalizability of this algorithm on heterogeneous datasets, coming from different populations: the test subsets of OPHDIAT, OphtaMaine (France, general population), RIADD (India, general population) and ODIR (China, general population). Since fundus photographs were acquired using different cameras in each dataset, there is a variability in terms of collected imaging data (see Fig. 1). To allow device-independent analysis, the size and the appearance of fundus photographs were normalized. Moreover, each dataset was annotated for a different set of ocular anomalies, thus label vocabularies and interpretations vary. In order to unify the ground-truth annotations, the annotation of each dataset was analyzed and converted into the ODIR annotation class system: Normal, Diabetes, Glaucoma, Cataract, AMD, Hypertension, Myopia and Other anomalies.

The SD network, trained on the OPHDIAT training subset, achieved a mean per-disease AUC (mAUC) of 0.9571 on the OPHDIAT test subset. However, much smaller mAUC values were obtained on the OphtaMaine, RIADD and ODIR test subsets (mAUC < 0.9, see Table 2). This indicates the limited generalizability of the SD network. In details, the ROC curves in Fig. 2 show that a performance decrease is observed for each disease category when the test subset comes from a different population than OPHDIAT. For all the test subsets, the Hypertension and Other categories were the most difficult to detect: this outcome could be explained by the relatively low number of examples for Hypertension and the diversity of anomalies in the Other class.

We hypothesized that training a DL algorithm jointly on multiple datasets, from distinct populations, would result in improved generalizability compared to the SD network. Therefore, Multiple-Dataset (MD) networks were trained using training subsets from all $K=4$ datasets (the joint dataset) or from $K=3$ datasets. For each dataset other than OPHDIAT, the MD networks outperformed the SD network when the training subset of the considered dataset was used for training the MD network (Table 2). In more details, the ROC curve comparison between the SD network and the MD network trained on all $K=4$ datasets for multi-disease detection on the OphtaMaine, the RIADD and the ODIR test subsets in Fig. 2 revealed that the AUCs are consistently higher for the MD network. In fact, the Wilcoxon test showed that there is a significant difference between the AUCs of the SD network and the AUCs of the MD network ($p=0.0058$, see Table 3). This indicates that jointly training on multiple datasets improves performance, but does it improve generalizability?

Despite the fact the MD networks showed good performances on data that have the same distribution as the training data, the performances suffered when the MD networks were tested on data very different from the training data. Leave-one-dataset-out experiments ($K=3$) revealed that performance on a test subset is significantly lower when the associated training and validation subsets are left out than when they are included ($p<0.0001$, see Table 3).

Therefore, this study suggests that developing a DL algorithm that can generalize well to unseen data coming from different populations is very challenging. Despite the improvement of performances using the MD network, it is still not able to generalize well to data that is very different from the training data. A possible explanation is that there is a variability in interpreting fundus photographs between the datasets. In fact, the label scope of each dataset is different as it depends on the screening purpose for which the dataset has been collected. In addition, even if a label is shared between two datasets, its definition may vary due to different annotation criteria. This could also be partly linked to the mismatch in the readers’ backgrounds (e.g. liberal for OphtaMaine and hospital practice for OPHDIAT). Also, the anomaly patterns may differ from one population to another: the location, shape and aspect of lesions may not be the same. The performances of the DL algorithm could as well be affected by the frequency of each anomaly in each dataset. For instance, since OPHDIAT is issued from a diabetic population, the DR is the most common pathology in the OPHDIAT dataset. In contrast, since OphtaMaine is issued from a general population, the glaucoma is the most common pathology in the OphtaMaine dataset. Finally, there is a possible variation in terms of collected imaging data which may affect the performances of the algorithm.

When compared to training a network on a single dataset, typically the large development set of currently commercialized systems, the MD network has three advantages: first, the size of the training data is increased (although not necessarily by a large factor); second, knowledge from multiple experts is integrated into a single model; and third, the combined training data better covers the variability of disease phenotypes across the World. Therefore, the Multi-Dataset learning strategy is efficient for training and inference. Nevertheless, it does not guarantee improved generalizability, so care should still be taken to evaluate the algorithms on various test datasets.

In summary, this study highlights the importance of assessing the generalizability of the DL algorithm. For this purpose, the ODIR annotation class system was proposed as unified classification, due to the lack of internationally recognized classification. The results showed that the SD network, trained on a single large dataset, generalizes poorly to new data which are very different from the training data. To remedy this problem, we proposed the MD algorithm which significantly improved the performances on new data. This strategy could be integrated in a learning scenario on multi-center health data. In this context, DL users (clinical centers) can largely benefit from participating in the enrichment of DL systems, since the performances will be significantly higher if they do. In future work, we will develop and assess distributed and secure DL solutions for multi-center training of eye pathology screening algorithms (LabCom ADMIRE project). Solving these challenges will pave the way for the large-scale deployment of DL systems and for the screening of many diseases.

## Methods

The methods were performed in accordance with relevant guidelines and regulations and approved by the French CNIL (National Information Science and Liberties Commission—approval #2166059). It followed the MR-004 reference methodology that provides a framework for non-interventional research involving health data of a public interest nature, carried out in the context of research involving the human person for which the data subject does not object to participating after having been informed. For the two public datasets used in this study (RIADD and ODIR), we followed instructions by the data manager, given on the dataset webpages. Informed consent was obtained from all subjects above 18. For OPHDIAT and OphtaMaine, some subjects are under 18: informed consent was obtained from a parent or legal guardian.

### Datasets

#### OPHDIAT dataset

The OPHDIAT screening network is a telemedical network created in Île-de-France, France. It focuses on diabetic retinopathy screening in a French diabetic population. Details on the OPHDIAT screening network are presented in^30^. The free-form screening reports originally written by the OPHDIAT ophthalmologists were retrospectively examined by a retina specialist: the purpose was to determine the presence or absence of 41 anomalies (pathologies or pathological signs) in each eye of each patient. These binary labels were consolidated with the structured information about the most prevalent pathologies originally given by the OPHDIAT ophthalmologists. Since the OPHDIAT screening network is specialized in detecting diabetic retinopathy, ophthalmologists may not have reported all of their findings. As a result, a retina specialist carefully reviewed normal fundus photographs to confirm that there were no anomalies. In this study, a selection of 42,990 screening examinations (corresponding to 37,141 diabetic patients and 77,827 fundus photographs) ($P$) was analyzed by the retina specialist, of which 16,955 images were labeled as “normal” and 60,872 were labeled as “anomalous”, that is, images showing signs of at least one of the 41 anomalies (pathologies or pathological signs).

Following common practice, the selected dataset ($P$) was divided into a training subset ${P}_{Train}$ (80% of $P$), used to optimize the model’s weights; a validation subset ${P}_{Validation}$ (10% of $P$), used to decide when to stop the optimization process and select the best model; and a test subset ${P}_{Test}$ (10% of $P$), used to evaluate the performance of the model. These subsets do not intersect: all fundus photographs from the same patient were assigned to the same subset. Patients were assigned to these subsets in such a way that the frequency of each anomaly is approximately the same in each subset; this was not always possible for rare anomalies. Aside from this criterion, assignment to subsets was done at random.

#### OphtaMaine dataset

OphtaMaine is a private screening network based in the Le Mans region, France. It targets a more general population to detect all eye pathologies. Details on the OphtaMaine screening network are presented in^30^. All examination records performed in OphtaMaine from 2017 to 2019 were included in this study, with the exception of those labeled “poor quality”. As a result, a total of 8131 examinations (17,120 fundus photographs) deemed of sufficient quality by OphtaMaine’s ophthalmologist were considered (dataset $A$). Like in OPHDIAT, the free-form comments were retrospectively evaluated to group examinations by types of anomalies (pathologies or pathological signs).

Following^30^, the dataset ($A$) was divided as follows: a training subset, ${A}_{Train}$(40% of $A$); a validation subset, ${A}_{Validation}$ (10% of $A$); and a testing subset, ${A}_{Test}$ (50% of $A$). These subsets do not intersect: all fundus photographs from the same patient were assigned to the same subset. Patients were assigned to these subsets in such a way that the frequency of each anomaly is approximately the same in each subset. Besides this criterion, assignment to subsets was made at random.

#### RFMiD dataset

The Retinal Fundus Multi-disease Image Dataset (RFMiD)^36^ is a publicly available retinal image dataset, as part of Retinal Image Analysis for multi-Disease Detection (RIADD) Challenge^37^, organized in conjunction with IEEE International Symposium on Biomedical Imaging (ISBI-2021), Nice, France. It enables the development of methods for automatic ocular disease classification of frequent diseases along with the rare pathologies. It comprises 3200 fundus images acquired using three different fundus cameras: Topcon 3D OCT-2000, Kowa VX-10_*α*_, and Topcon TRC-NW300, all of them centered either on the macula or optic disc. These photographs are taken from Indian people who went to an eye clinic because they were concerned about their eye health. Initially, two ophthalmologists independently read all the images. Based on a thorough examination of the participants’ clinical records and visual fields, a reference standard for the presence of different diseases was assigned. If a fundus image reveals the presence of numerous diseases, the image is labeled with multiple labels. Following the ophthalmologists’ initial labeling of fundus photographs, the project team’s leader double-checked and confirmed or corrected the labels with input from both ophthalmologists when discrepancies in diagnostic assessments were discovered, resulting in adjudicated consensus for the labels^32^. The RFMiD annotations comprises screening of fundus photographs into normal and abnormal (comprising of 45 different types of diseases/pathologies) categories. It also includes the classification of fundus photographs into 45 different categories^32^.

Following^32^, the full dataset, $R$, is divided into three subsets: a training subset, ${R}_{Train}$ 60% (1920 images); a validation subset, ${R}_{Validation}$ 20% (640 images); and a testing subset, ${R}_{Test}$ 20% (640 images).

#### ODIR dataset

Ophthalmic Image Analysis-Ocular Disease Intelligent Recognition (OIA-ODIR) is a multi-disease fundus image dataset^33^. It is available as part of the Ocular Disease Intelligent Recognition challenge^29,38^. It is intended to reflect a "real-life" set of patient data collected by Shanggong Medical Technology Co., Ltd. from various hospitals and medical centers in China. It comprises 10,000 fundus photographs acquired from left and right eyes of 5,000 Chineese patients, using different cameras such as Canon, Zeiss and Kowa. It is annotated by trained human readers with quality control management: patients are classified into eight labels based on both eye images and additionally patient age. The annotations consist of normal (N), diabetes (D), glaucoma (G), cataract (C), AMD (A), hypertension (H), myopia (M) and other diseases/abnormalities (O).

Following^33^, the full dataset, $I$, is split into three subsets: the training set (3500 patients), the off-site test set (500 patients) and the on-site test set (1000 patients). In this work, we used the training set ${I}_{Train}$ for training deep networks, the off-site test set ${I}_{Validation}$ as validation subset for model selection and the on-site test set ${I}_{Test}$ as testing subset for evaluating the generalization ability of the deep network.

### Development of a single-dataset network for multi-disease detection

The Single-Dataset network (SD) is a classifier that directly maps a fundus photograph to ODIR labels: D, G, C, A, H, M and O. Since a fundus photograph can be associated to multiple labels simultaneously, this is a multi-label classification problem. Consequently, it was trained using the binary cross entropy loss. We remind that the SD network is trained using the training and validation subset of the largest dataset, OPHDIAT.

The first development step was to tune hyperparameters. Based on benchmark analysis of popular ImageNet classification deep CNN architectures, we considered eight CNN architectures which were among the best performing networks on ImageNet^39^: Efficientnet-b0, Efficientnet-b5^40^, tf_efficientnet_b5_ns, tf_efficientnet_b0_ns^34^, Efficientnetv2_l, tf_efficientnetv2_xl_in21k^41^, swin_large_patch4_window12_384^42^, and vit_large_patch16_384^43^. These CNNs were trained using different augmentation strategies: Horizontal flip, Randaugment^44^ and the augmentation proposed by Kamatalab team^45^ which ranked first on the off-site challenge leaderboard of RIADD^28^. We denote the latter by Kamatalab_augment. We used AdamW optimizer with weight decay of 0.0005. We also performed an exponential moving average with the momentum of 0.999. We used a learning rate of 0.001. The CNN architecture and the augmentation strategy were chosen through a ROC analysis conducted in the OPHDIAT validation subset. Precisely, the AUC was calculated independently for each anomaly class of the ODIR annotation system and then the average per-class AUC (denoted mAUC) were computed. The hyperparameters (CNN architecture and augmentation strategy) maximizing the mAUC were retained; the same hyperparameters were used in the following experiments.

Table 4 reports the mAUC on the validation subset of OPHDIAT for the 8 considered CNNs and for the three studied augmentation strategies using the SD network. The "tf_efficientnet_b5_ns" with Randaugment showed the best performances for anomaly detection on the OPHDIAT validation subset. Therefore, tf_efficientnet_b5_ns with Randaugment was used as a backbone in all our experiments.

**Table 4:** mAUC computed on the validation subset of the OPHDIAT dataset for the SD network.

	Horizontal Flip	Randaugment	Kamatalab_augment
Efficientnet-b5 (456 × 456)	0.9449	0.9117	0.9383
tf_efficientnet_b0_ns (224 × 224)	0.9555	0.9503	0.9472
Efficientnet-b0 (224 × 224)	0.9557	0.9549	0.9483
tf_efficientnet_b5_ns (456 × 456)	0.9616	**0.9633**	0.9623
tf_efficientnet_v2_xl_in21k (448 × 448)	0.9600	0.9503	0.9576
efficientnet_v2_l (448 × 448)	0.9267	0.8341	0.9421
vit_large_patch16_384 (384 × 384)	0.7091	0.6391	0.6582
swin_large_patch4_window12_384 (384 × 384)	0.5938	0.6460	0.5798

Significant values are in bold.

### Development of a multiple-dataset network for multi-disease detection

The Multiple-Dataset network (MD) is a single detector that is trained on $K$ datasets ${d}_{1}, \dots , {d}_{K}$. In this work, we study different scenarios. In each scenario, we train a network on a different combination of datasets. For validation, we compute the mAUC on the validation subset of each dataset independently and then compute the average. The MD network showing the best average mAUC score is then selected.

For optimizing the joint training on multiple datasets, we study three different strategies. The first strategy consisted of assigning an equal weight to each dataset during training (MD Equal). The second strategy involved assigning a weight proportional to the size of each dataset during training (MD Proportional). Finally, the third strategy consisted of assigning a weight which corresponds to the logarithmic scale of the size of each dataset during training (MD Uniform). The MD strategy was then selected based on the mAUC computed on the validation subset of the four datasets.

The same hyperparameters (MD strategy, as well as CNN architecture and augmentation strategy determined previously) are used in all MD experiments.

Table 5 reports the mAUC on the validation subset of each dataset for the MD network using the aforementioned MD strategies. This table reveals that the MD Equal showed the best results for most considered datasets. Thus, this strategy was selected in all our experiments for the MD network.

**Table 5:** mAUC on the validation subset of each dataset for the MD Equal, the MD Proportional and the MD Uniform strategies using tf_efficientnet_b5_ns with Randaugment.

	OPHDIAT	OphtaMaine	RIADD	ODIR
MD equal	0.9454	0.9151	0.9474	0.9092
MD proportional	0.9262	0.84505	0.8637	0.8546
MD uniform	0.9442	0.91115	0.9331	0.9120

### Main outcome measure

The SD and MD networks were evaluated using AUCs calculated independently for each test dataset and for each anomaly class of the ODIR annotation system (except H and C for OphtaMaine, and H for RIADD—see Results Section). A total of 25 AUCs were thus computed for each network (4 test datasets $\times$ 7 classes $-$ 3 exclusions).

To assess the generality of the MD network, a leave-one-dataset-out experiment was conducted: each of the four datasets, in turn, was set fully aside for testing, i.e. its training and validation subsets were ignored. Next, a statistical evaluation was performed to compare the 25 test AUCs obtained when the corresponding training/validation datasets are ignored to those obtained when they are used. Since the AUC differences were not normally distributed and the number of paired samples (25) is less than 30, the paired samples Wilcoxon test^35^ was applied, instead of the usual paired samples t-test. Note that, in the leave-one-dataset-out experiment, each dataset is excluded from a single 3-tuple of datasets, but it is included in three: the one leading to the lowest mAUC was used in the comparison.

Furthermore, to compare the generality of the SD and MD networks, we also applied the paired samples Wilcoxon test to investigate whether the test AUCs obtained with the SD network are significantly different from those obtained with the MD network, when the corresponding training and validation datasets are included.

## Data Availability

The data that support the findings of this study may be available from DR screening program of OPHDIAT^©^ and private screening program of OphtaMaine^©^, but restrictions apply to the availability of these data. These data, or a test subset of them, may be available subject to ethical approvals. The ODIR and the RIADD datasets are publicly available at the following URLs: RIADD dataset: https://riadd.grand-challenge.org/. ODIR dataset: https://github.com/nkicsl/OIA-ODIR.

## Code availability

The Machine learning models were developed using Pytorch Image models available at: https://github.com/rwightman/pytorch-image-models. Custom code based on Pytorch was specific to our computing infrastructure and mainly used for data input/output.
